# Exploring the biological mechanisms of obesity affecting breast cancer from a multidimensional perspective

**DOI:** 10.1007/s12672-025-03838-9

**Published:** 2025-11-21

**Authors:** Yingying Rao, Suyuan Wang, Xianglin Liu, Changhua Shao, Hengyu Li

**Affiliations:** 1 Department of Breast and Thyroid Surgery, The First Affiliated Hospital of Naval Medical University, Shanghai, 200433 China; 2 The First Affiliated Hospital of Naval Medical University, Shanghai, 200433 China

**Keywords:** Obesity, BMI, High-fat diet, Breast cancer, Tumor immune microenvironment, Intestinal microecology, Circadian homeostasis, Biological mechanisms

## Abstract

Obesity, a global health challenge, significantly contributes to breast cancer pathogenesis through chronic inflammation and metabolic dysregulation. This review summarizes the biological mechanisms through which obesity influences the development and progression of breast cancer, focusing on endocrine regulation, intercellular communication, gut microbiota interactions, obesity-associated genetic polymorphisms, and circadian rhythm homeostasis. The aim is to provide a theoretical foundation for the diagnosis and treatment of obesity-related breast cancer.

## Introduction

Obesity is the excessive accumulation of body fat, defined by the World Health Organization (WHO) as a body mass index (BMI) ≥ 30 kg/m² [1]. According to this definition, research shows that the global prevalence of obesity continues to rise, and by 2025, it is projected to reach 18% among men and over 21% among women [2]. Obesity has been established as a significant risk factor associated with the development and progression of cardiovascular diseases, type 2 diabetes, and various malignancies—including breast cancer, colorectal cancer, renal cancer, and prostate cancer, among others—thereby emerging as a widespread concern [3, 4].

Breast cancer, one of the most common malignant tumors globally, poses a serious threat to both the physical and mental health of patients and imposes a significant social and economic burden. This review reveals that the biological mechanisms linking breast cancer and obesity are complex, involving not only lipid metabolism but also gut microbiota dysfunction and disruption of circadian rhythm homeostasis.

Most studies have shown that obesity is positively associated with the risk of postmenopausal breast cancer, while the association with premenopausal breast cancer seems to be controversial [5]. In addition, the specific associations between obesity and different subtypes of breast cancer remain poorly understood. In obese postmenopausal women, increased aromatization-derived estrogen leads to elevated estrogen exposure, which stimulates the proliferation of mammary epithelial cells and contributes to a higher lifetime risk of ER⁺ breast cancer [6]. In contrast, obese premenopausal women more frequently exhibit suppression of the hypothalamic-pituitary-ovarian (HPO) axis and prolonged anovulatory cycles, resulting in reduced cumulative hormone exposure [7]. Furthermore, the association between obesity and breast cancer incidence in premenopausal women appears to be unique to triple-negative breast cancer (TNBC) [8], although the underlying mechanisms remain incompletely elucidated. Studies indicate that obesity may promote the development and progression of TNBC through mechanisms involving immune microenvironment modulation, fatty acid metabolism, and insulin signaling pathways [8]. For example, obesity can enhance the secretion of immunosuppressive cytokines such as IL-10 and increase levels of certain free fatty acids, thereby influencing macrophage polarization and anti-tumor immune responses [8, 9]. Insulin-driven systemic metabolic alterations may also contribute to breast cancer pathogenesis, particularly in TNBC [8]. It is plausible that in premenopausal breast cancer, obesity may predominantly operate through the aforementioned non-hormonal mechanisms. We conclude that the influence of obesity on breast cancer is heterogeneous across menopausal status and molecular subtypes, involving context-dependent mechanisms such as hormone receptor signaling, immune infiltration, and metabolic adaptations. Therefore, further investigation of the biological mechanisms by which obesity influences breast cancer development is essential for improving the management and treatment of obesity-related breast cancer and for reducing the societal burden of obesity-related cancers.

## Endocrine mechanisms

### Adipose tissue-regulated adipokines

Adipose tissue, an endocrine organ, not only stores fat but also influences the synthesis and secretion of various adipokines and exerts widespread effects on systemic metabolism [10]. Studies indicate that obesity is characterized by a state of chronic low-grade inflammation and dysregulation of various adipokine levels. For instance, dysfunctional adipose tissue elevates the production of the pro-inflammatory mediator leptin while reducing levels of adiponectin, an adipokine with anti-inflammatory and anti-tumor properties [11, 12]. Moreover, adipocytes can also secrete pro-inflammatory cytokines such as TNF-αand IL-6, which are involved in inflammation-related carcinogenic processes [11]. Below, we will examine the relationships of two prominent adipokines—adiponectin and leptin—with breast cancer.

### Effects of leptin and adiponectin on breast cancer

Leptin (LEP) is a pleiotropic peptide hormone, encoded by the obesity (Ob) gene, that acts by binding to its receptor (ObR) or interacting with growth factors and the estrogen receptor (ER), and is involved in biological processes such as appetite regulation and energy balance [13]. To date, the characterization of leptin’s role in breast cancer remains somewhat controversial and appears to be associated with body weight and women’s menopausal status, and to have an impact on signaling pathways involved in different molecularly typed breast cancers [16]. For instance, a systematic review suggested that LEP may serve as a potential marker for cancer risk assessment in postmenopausal obese women, while no significant association was found with breast cancer risk in premenopausal women [14]. Another multicenter study reported that the effect of BMI on breast cancer risk in premenopausal women is complex [15].

LEP regulates multiple key signaling pathways. For instance, the importance of STAT3 in promoting glycolysis through transcriptional regulation has been demonstrated in proliferating breast cancer cells [17]. Inhibition of JAK/STAT3 signaling suppresses self-renewal of breast cancer stem cells (BCSCs) and downregulates key lipid metabolic genes such as carnitine palmitoyltransferase 1B (CPT1B), which encodes a rate-limiting enzyme in fatty acid β-oxidation (FAO). Adipocyte-derived leptin (LEP) is critical for activating the JAK/STAT3-FAO axis in BCSCs. LEP promotes stemness and chemoresistance by upregulating STAT3-induced CPT1B expression and enhancing FAO activity [18].

Additionally, LEP stimulates breast cancer cell proliferation and migration via MAPK pathway activation [19], and modulates the PI3K/AKT pathway to facilitate epithelial–mesenchymal transition (EMT), migration, and angiogenesis [20]. These studies suggest that targeting key mediators of these pathways may offer a potential therapeutic strategy for breast cancer.

Furthermore, studies have shown that LEP affects T-cell differentiation and function through modulation of inflammatory mediators (IL-2, IFN-γ, etc.) [21, 22]. Specifically, LEP has been shown to enhance CD4 + T cell activation and promote a shift in CD4 + T cell polarization from a Th2 toward a Th1 phenotype [16]. Additionally, LEP mediates macrophage functional polarization via mast cell signaling: leptin-deficient mast cells can redirect macrophage polarization from the M1 to the M2 phenotype [23]. These findings highlight the complex crosstalk between LEP and immune cells; however, the relevance of these interactions in the context of breast cancer remains to be elucidated [24].

In conclusion, LEP promotes breast cancer progression through multiple mechanisms; however, certain aspects, such as how its interaction with immune cells influences tumor development, remain not fully elucidated. Further investigations using both in vivo and in vitro models are necessary to establish the mechanistic basis for precision therapy in breast cancer.

Adiponectin is an adipokine primarily secreted by adipocytes, which affects target tissues through its receptor (AdipoR) and exerts anti-inflammatory, insulin-sensitizing, and energy metabolism-regulating effects [25, 26]. Moreover, unlike the pro-cancer effects of many adipokines, adiponectin exerts inhibitory effects on breast cancer. Several meta-analyses have demonstrated an association between reduced circulating levels of adiponectin and an increased risk of breast cancer, suggesting that it may serve as a biomarker for identifying individuals at high risk of developing breast cancer [27, 28].

Currently, a growing number of therapeutic strategies aimed at enhancing adiponectin activity have emerged [25], offering new potential for the treatment of obesity-associated breast cancer. For instance, the classic antidiabetic drug metformin, described as an adiponectin agonist, has been shown to increase serum adiponectin levels and has gained attention in oncology for its demonstrated antitumor effects, particularly in breast cancer [29]. In addition to metformin, the glucagon-like peptide-1 (GLP-1) receptor agonist liraglutide has been shown to suppress breast cancer cell activity and increase mRNA levels of adiponectin and its receptors in obesity-associated breast cancer [30]. Several other small-molecule compounds have also demonstrated adiponectin-like effects and exhibit certain anti-tumor biological activities [25]. The mechanisms of these agents offer promising avenues for breast cancer treatment, and suggest that circulating adiponectin levels and AdipoR expression may serve as potential biomarkers for identifying patient populations likely to benefit from such therapies.

A review of the literature indicates that the cancer-inhibitory effects of adiponectins are mediated through multiple pathways [25]. For instance, these include targeting the Wnt and Akt pathways to inhibit MDA-MB-231 cell growth and proliferation [31], inducing apoptosis in MCF-7 cells through cell cycle regulation [32] and mechanisms that inhibit leptin-mediated cell proliferation by regulating the mRNA levels of LEP and ObR [33].

Additionally, adiponectin exhibits immunomodulatory effects by promoting the polarization of macrophages toward the M2 anti-inflammatory phenotype [34]. However, due to limited current evidence, further investigation is warranted to elucidate how adiponectin influences the breast cancer immune microenvironment through the regulation of immune cells.

As previously mentioned, although LEP is known to promote breast cancer progression by regulating multiple key signaling pathways, its association with breast cancer risk appears heterogeneous between pre- and post-menopausal women. Since LEP can interact with the ER, we suggest that this discrepancy may be related to interactions with hormone levels across different age groups. Furthermore, although the tumor-suppressive effects of adiponectin are supported by meta-analyses, its specific mechanisms of regulating immune cells—such as macrophage polarization—remain poorly understood. Future studies should take into account molecular subtypes and menopausal status to elucidate the complex characteristics of adipokine actions.

### Association of insulin resistance, hyperinsulinemia and obesity-related breast cancer

A growing body of research has demonstrated a strong association between obesity, T2D, and breast cancer [35–37]. Further evidence suggests that obesity and T2D increase the risk of breast cancer, reduce quality of life, and decrease survival rates [38, 39].

Insulin resistance, hyperinsulinemia and elevated blood glucose are known to be major pathophysiological features of obese and T2D patients, and studies have shown that these metabolic disorders promote the development of breast cancer [40, 41].

Mechanistically, studies have demonstrated that the PI3K/Akt signaling pathway mediates the biological effects of insulin and insulin-like growth factor-1 (IGF-1), and is aberrantly activated in diabetes/impaired glucose tolerance (IGT), obesity, and breast cancer, suggesting a potential link among these comorbidities [42]. In a study using spontaneously obese rats with impaired glucose tolerance (WNIN/GR-Ob rats) to explore related molecular mechanisms, elevated expression of signaling molecules including IGF-1, IGF-1R, PI3 kinase, pAkt, and ER was observed in mammary tumor tissues of obese rats compared to lean rats, promoting mammary cell proliferation and dissemination [43]. These findings suggest that targeting the PI3K/Akt signaling pathway and the insulin metabolic regulatory system may hold significant clinical implications for patients with such comorbidities, though further research is warranted to elucidate the underlying mechanisms and identify therapeutic targets. Clinical attention should be directed to this metabolic disorder, and health education along with comprehensive treatment plans should be developed for patients with this comorbidity.

## Inter-cellular communication networks

### Effect of obesity-associated adipocyte exosomes on breast cancer cells

Exosomes are nanoscale vesicles produced by cells in response to environmental stimuli or self-activation, playing a crucial role in intercellular communication [44, 45]. In obese populations, studies have demonstrated that adipocyte-derived exosomes contribute to the progression of cancers, including breast cancer, by affecting tumor EMT and promoting an aggressive phenotype [46–48]. This phenomenon was observed in a mechanistic study using a high-fat diet (HFD)-fed mouse model, where HFD-induced alterations in exosome content mediated molecular changes in phenotypic characteristics of TNBC cells, promoting EMT and metastasis, potentially involving alterations in the regulation of key signaling pathways such as Rac1 [49]. Additionally, other studies have shown that vesicles isolated from adipocytes can alter matrix metalloproteinase (MMP) activity, enhancing the migration and invasion of MCF-7 and MDA-MB-231 cells [50–52].

The biological mechanisms through which adipocyte-derived exosomes influence breast cancer development remain poorly understood, and further studies are required to explore additional therapeutic strategies. For instance, exosomes may serve as valuable biomarkers for breast cancer detection, while the emergence of artificially produced therapeutic exosomes may enable targeted drug delivery and therapeutic sensitization [53].

### Shaping of the tumor immune microenvironment by adipocyte interactions with other cells

#### Formation of the inflammatory microenvironment

**Fig. 1:**
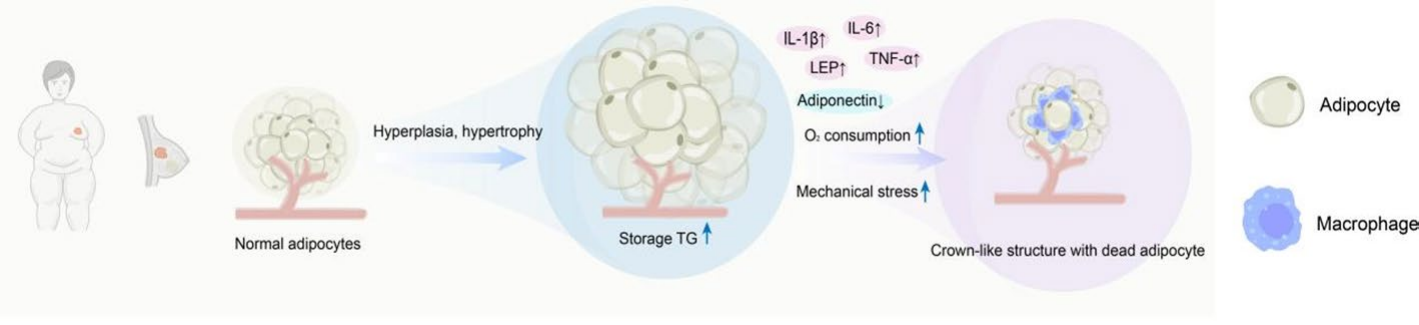
Schematic illustration of the formation of the crown-like structure

In obesity, adipocytes undergo hypertrophy and hyperplasia to store increased triglyceride levels [54]. This leads to adipose tissue dysfunction due to factors such as mechanical stress and increased local oxygen demand, resulting in the increased release of chemokines and inflammatory factors [55–57]. As hypertrophic adipocytes die, additional pro-inflammatory factors are released, leading to macrophage recruitment and a shift in polarization, forming specialized crown-like structures (CLS) around dead adipocytes and sustaining a pro-inflammatory environment (Fig. 1) [58]. Mechanistic studies [60] have also revealed that saturated fatty acids (SFAs) derived from adipocytes induce the expression of pro-inflammatory factors such as TNF-αand IL-6 through Toll-like receptor 4 (TLR4). In turn, these macrophage-derived pro-inflammatory factors act on adipocytes to promote lipolysis. The formation of CLS can be viewed as a hallmark of obesity-induced adipose tissue inflammation. Adipocytes and macrophages engage in complex crosstalk, interacting via secretory factors and creating a vicious cycle of inflammatory response during the progression of obesity [60]. Additionally, hypertrophied adipocytes promote the secretion of inflammatory factors by influencing the expression of LEP and adiponectin [55]. A mechanistic study exploring the molecular links between immune cells, adipocytes, and breast cancer using a 3D co-culture model found that macrophage-conditioned medium promotes the release of pro-inflammatory factors, such as IL-1β and TNF-α, from adipocytes, increasing the expression of matrix metalloproteinases in the microenvironment and enhancing the invasive capacity of breast cancer cells [61]. Other studies also highlight that paracrine activity between macrophages and adipocytes plays a critical role in maintaining the inflammatory microenvironment in obese patients [62, 63]. Moreover, inflammatory factors produced through intercellular interactions are linked to poor prognosis in breast cancer [64]. This evidence suggests that regulating the secretion of inflammatory molecules from immune cells, such as macrophages, can improve the immune microenvironment in patients with obesity-associated breast cancer and mitigate the tumor-promoting effects of inflammatory conditions.

Additionally, the increased release of adipokines, such as leptin and resistin, can trigger inflammatory signaling in various immune cells [65]. Transcriptomic data from breast cancer patients also suggest that the obese population harbors numerous genes associated with inflammation and immune cell communication [66].

In the obese state, adipose tissue undergoes dysfunction, accompanied by alterations in the expression of multiple cytokines and the formation of a chronic inflammatory microenvironment. In the pro-inflammatory context, macrophages are recruited around dying or dead adipocytes, ultimately leading to the formation of the crown-like structure.

#### Construction of immunosuppressive microenvironment

The formation of the tumor microenvironment (TME) involves interactions between tumor cells, immune cells, and stromal cells, making it a key target for cancer therapy [67]. In recent years, there has been an increase in studies examining the effects of obesity on the TME in breast cancer, particularly its impact on the development of the immunosuppressive microenvironment.

Macrophages play a crucial role in shaping the immunosuppressive microenvironment in obesity. On one hand, various cytokines produced by macrophages interacting with adipocytes contribute to a chronic low-grade inflammatory state, impairing immune cell function and promoting tumor progression [68]. Studies also show dysregulation of the M1/M2 macrophage ratio in tumors of obese mice, which is associated with poor prognosis [69]. Additionally, obesity causes M1-type macrophages to upregulate PD-L1 in TNBC, positively correlating with metabolic complications in patients [70]. On the other hand, preclinical models suggest that cytokines produced by macrophages in obese breast cancer patients can induce a tumor stemness phenotype, further promoting tumor progression [71].

Adipose tissue expansion, coupled with altered CD8 + T cell ratios, creates an obesity-specific immune signature that facilitates tumor progression [72]. The increased release of adipokines, such as LEP, enhances LEP signaling, which prompts T cells to overexpress PD-1, thereby mediating T cell depletion and immune decay [73, 74]. Studies have confirmed that the abundance of LEP is related to the proliferation of CD8 + T, CD4 + Th1 cells and Tregs [75]. Tregs are known to play a crucial role in suppressing NK and CD8 + T cell-mediated anti-tumor immune responses [76].

In obesity, both the number and activity of NK cells are impaired. Similarly, elevated LEP levels alter signaling at the NK cell LEP receptor and affect the production of immunomodulatory factors [77, 78].

Overall, obesity exerts a complex effect on the tumor immune microenvironment. The interaction between adipocytes, immune cells, and the cytokines produced by both contributes to the development of an immunosuppressive microenvironment, primarily characterized by chronic inflammation and suppression of anti-tumor immunity. Improving the immune microenvironment and regulating or reversing this immunosuppressive state is critical for the treatment of obesity-related breast cancer.

## Gut microbial community associations

The gut microbiota (GM) plays a crucial role in human metabolism and disease progression, and is strongly associated with nutrient absorption, immune homeostasis, and metabolic balance [79]. Increasing evidence suggests that the dietary composition in obese populations influences gut microbial homeostasis and is linked to several diseases, including breast cancer [79, 80].

**Fig. 2:**
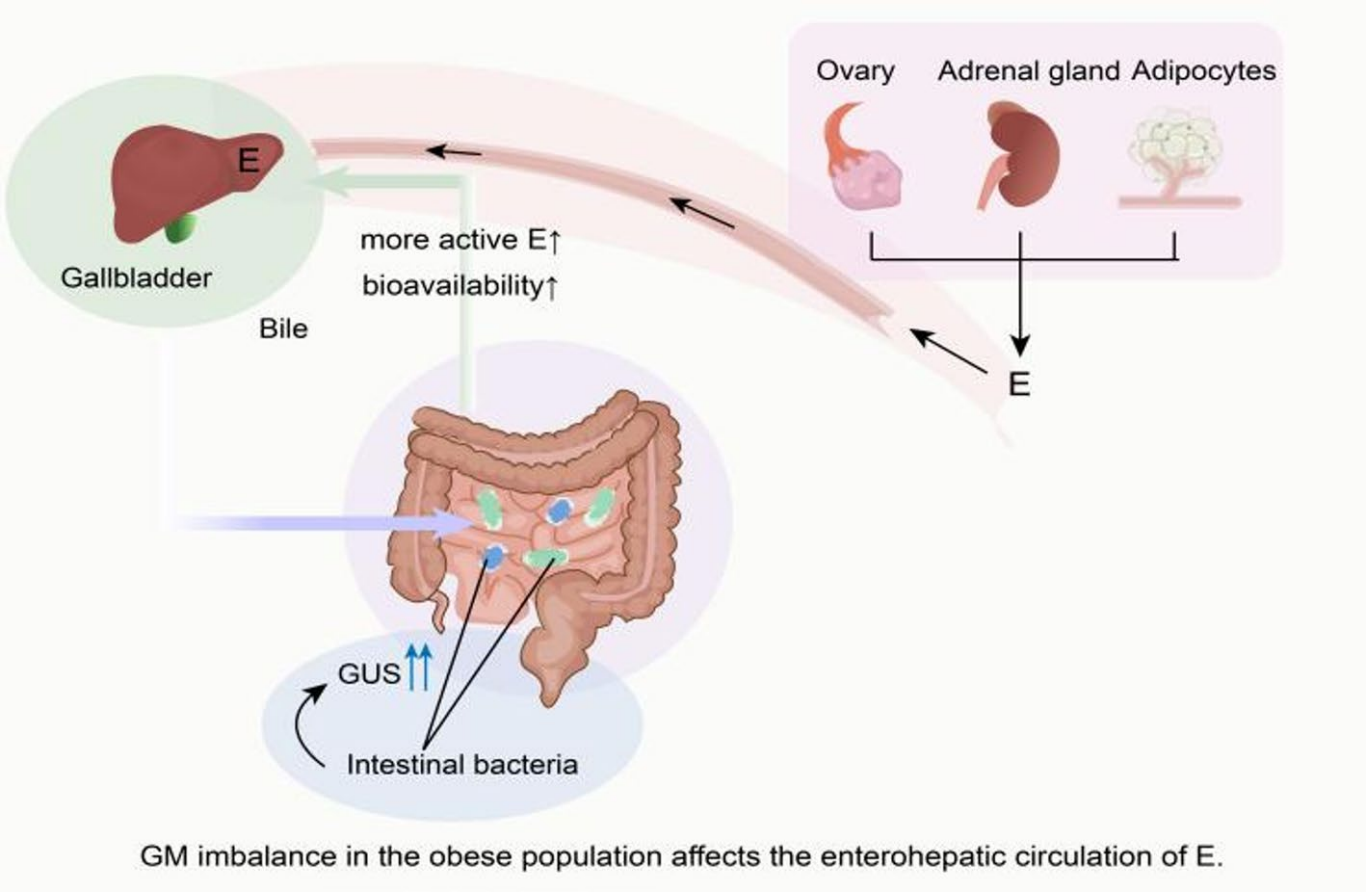
The effect of gut microbiota imbalance on the enterohepatic circulation of estrogen (E) in the obese population

We emphasize that the secondary effects of obesity-mediated gut dysbiosis may influence the initiation and progression of breast cancer. Studies indicate that GM diversity and abundance are altered in obese populations, characterized by a decrease in Bacteroidetes abundance and an increase in Bacillota abundance [81, 82]. This host-gut microecological imbalance may contribute to oncogenesis by modulating inflammation, immune cell function, and gene expression within the host [83, 84]. For instance, GM dysbiosis reduces short-chain fatty acid production and enhances pro-inflammatory signaling, thereby exacerbating obesity-associated chronic inflammation [85]. As previously noted, chronic inflammation promotes the formation of an immunosuppressive microenvironment in breast cancer, and the underlying mechanisms require further exploration. Additionally, GM imbalance is accompanied by changes in gut microbial metabolites, with β-glucuronidase (GUS) being a key mediator, as it has long been implicated in estrogen metabolism [86]. Increased GUS expression in obesity elevates estrogen levels in the bloodstream by regulating the enterohepatic recycling of estrogen, thereby enhancing estrogen bioavailability and increasing the risk of breast cancer (Fig. 2) [86, 87]. Another mechanistic study indicated that the microbiota exposed to a high-fat diet (HFD) releases abundant leucine, which activates the mTORC1 signaling pathway in myeloid progenitor cells, thereby promoting the infiltration of polymorphonuclear myeloid-derived suppressor cells (PMN-MDSCs) and contributing to poor clinical outcomes in breast cancer [88].

GM homeostasis also influences breast cancer treatment responses [89]. A mechanistic study showed that the efficacy of trastuzumab in HER2-positive breast cancer is regulated by GM [90], as demonstrated by the effect of antibiotic use on GM homeostasis, leading to reduced dendritic cell activation, IL-12p70 release, and decreased NK and T cell immune function post-trastuzumab treatment. Another mechanistic study suggests that gut bacteria or their metabolites enhance immune checkpoint blockade (ICB) efficacy in breast cancer patients by promoting CD8 + T cell stemness [91].

Further studies are needed to clarify the additional mechanisms by which obesity-induced GM dysbiosis may affect the development of breast cancer. A deeper understanding of the mechanisms by which the GM influences breast cancer progression and treatment response will facilitate the identification of clinically relevant responsive biomarkers and enable targeting of the GM. Interventions such as fecal microbiota transplantation (FMT) have been proposed for breast cancer patients. In addition to FMT, probiotic supplementation may enhance intestinal barrier function and represents another potential therapeutic strategy, particularly in overweight patients [92]. However, based on current evidence, we emphasize that the application of these therapies in breast cancer remains exploratory. Further large-scale, high-quality clinical studies are needed to establish optimal dosing, identify suitable patient populations, and evaluate long-term safety.

As mentioned previously, the secondary effects of GM imbalance may influence the initiation and progression of breast cancer, potentially involving estrogen metabolism, lipid metabolism, and immune regulation. However, specific mechanisms remain incompletely elucidated—such as which particular bacteria or metabolites play critical roles at which stages, and how they interact with breast cancer cells. Future investigations should employ multi-omics technologies to further unravel these complex mechanisms.

Estrogen is produced by the ovary, adrenal gland, and adipocytes, metabolized in the liver, and excreted into the intestine with bile. β-Glucuronidase (GUS) expressed by intestinal bacteria can affect the activity and bioavailability of estrogen. Gut microbiota imbalance in the obese population alters the expression or activity of GUS, thereby influencing the enterohepatic circulation of estrogen.

## Polymorphisms of obesity-related genes and breast cancer

Studies have indicated that polymorphisms in obesity-associated genes appear to be significantly correlated with a differential risk of breast cancer development [93].

The fat mass and obesity-associated (*FTO*) gene, located on the 16q12.2 region of the human chromosome, was the first identified gene to play a significant role in energy metabolism and body weight regulation, and is closely associated with cancer stem cell self-renewal, tumor invasion, and drug resistance mechanisms [94]. Studies have shown that single nucleotide polymorphisms (SNPs) in *FTO* are associated with the risk of various cancers, including breast cancer [94, 95]. For instance, the SNP rs9939609 in *FTO* is linked to estrogen receptor activity and the PI3K/Akt signaling pathway [96, 97]. Furthermore, *FTO* is involved in regulating key signaling pathways, including regulating the metabolic processes of breast cancer cells through the m6A methylation modification pathway, which influences tumor growth and proliferation [94]. Given *FTO’s* cancer-promoting effects, a variety of FTO inhibitors have been developed, and their therapeutic efficacy has been validated in tumor models [94]. For example, MO-I-500 significantly suppresses the survival and colony-forming ability of TNBC cells, demonstrating anti-tumor efficacy [94]. To our knowledge, several candidate FTO inhibitors have entered preclinical studies and early-phase clinical trials, and we anticipate their clinical translational potential for the treatment of breast cancer.

Polymorphisms in the LEP and LEPR genes are associated with an increased risk of breast cancer and more aggressive disease progression [98]. Studies indicate that variation in the promoter region of the LEP gene at position − 2548 is linked to elevated breast cancer risk, with this association being more pronounced in women with obesity [99]. Specifically, the LEP − 2548AA genotype confers a slightly higher risk of breast cancer compared to the − 2548GG genotype [99].

Mutations in the melanocortin 4 receptor (MC4R) gene, such as rs17782313 (T/C), are associated with monogenic forms of severe early-onset obesity and have been implicated in an increased risk of breast cancer [93]. However, a meta-analysis indicated that the rs17782313 variant is associated with colorectal cancer, but not breast cancer [100].

Additionally, Insulin Receptor Substrate-1 (IRS-1) is a key adaptor protein involved in insulin receptor signaling. The IRS-1 Gly972Arg variant constitutes a risk factor for insulin resistance, particularly in individuals with obesity [101]. This association raises the question of whether it may also contribute to obesity-associated breast cancer progression. However, available evidence indicates that the IRS-1 Gly972Arg polymorphism is not associated with breast cancer-specific or all-cause mortality [102]. Nonetheless, owing to the limited number of studies on this variant, this conclusion should be interpreted with caution.

In summary, the role of genetic polymorphisms associated with obesity in breast cancer development requires further validation, and many questions remain unresolved. These include how such polymorphisms interact with environmental factors to influence breast carcinogenesis; the effects of specific genetic variants across different breast cancer subtypes; how they affect tumor biological behavior in various contexts; and importantly, how established genetic risk markers can be translated into clinical practice.

## Role of biological clock disruption in obesity and breast cancer development

Circadian rhythms regulate various physiological processes, including energy metabolism and sleep-wake cycles, and are controlled by a set of biological clock genes essential for normal life activities [103]. The biological clock is an endogenous timekeeping system comprising a central biological clock in the suprachiasmatic nucleus (SCN) of the hypothalamus and a peripheral biological clock. The SCN synchronizes the phase of the peripheral biological clock via signaling and regulates endocrine rhythms and metabolic processes in the body [103].

A substantial body of evidence indicates a bidirectional relationship between the biological clock and obesity [109]. On one hand, disruption of circadian rhythms by genetic or environmental factors may increase the risk of obesity and lead to metabolic disorders, including insulin resistance [104, 105]. This concept has been supported not only in mouse models [106, 107], but also in populations with altered work schedules [105, 108]. On the other hand, disruption of circadian rhythms has been observed in mouse models of obesity [110], and the expression of biological clock genes in obese populations shows alterations [111, 112]. For instance, the study by Wang et al. reports that obesity-induced reduction of PPAR-γ mediates the downregulation of SLC1A5, which disrupts BMAL1 and other biological clock genes, leading to disturbance of the white adipose tissue (WAT) biological clock in both mice and humans [109]. PPAR-γ is primarily expressed in adipose tissue and plays a crucial role in lipid metabolism; its expression has been associated with various diseases, including breast cancer [109, 120].

Various biological clock genes involved in regulating circadian rhythms are associated with the development of breast cancer [113]. For instance, reduced expression of PER2, a key factor that negatively regulates circadian rhythms and is considered an oncogenic factor, may promote excessive proliferation of breast cancer cells [114]. PER may also regulate ER expression in collaboration with the BRCA1 protein [119]. Additionally, BMAL1, a key transcription factor of the biological clock, influences the antioxidant process and exerts pro-cancer effects by regulating Nrf2 [115]. Biological clock genes also regulate inflammatory processes, impacting immune cell function and the breast cancer microenvironment [113]. For instance, circadian rhythm disruption promotes neutrophil-driven inflammation [116], causes an imbalance between M1 and M2 type macrophages [117], and alters the expression of EMT-related genes, thereby increasing breast cancer cell invasiveness [118]. These mechanisms suggest that disruption of the biological clock plays a critical role in breast cancer progression.

Disruption of the biological clock increases the risk of metabolic disorders and obesity, and is associated with breast cancer progression. Conversely, obesity can disrupt the expression of circadian clock genes and may create a favorable environment for breast cancer cell growth and proliferation. Together, these observations illustrate the complexity of the circadian rhythm–obesity–breast cancer axis. Overlapping molecular mechanisms may underlie their associations; however, current evidence remains limited, and these relationships are likely indirect or speculative rather than representing direct causation. These observations highlight the need for further targeted research to clarify the nature and interactions within this network.

## Conclusions and outlook

The biological mechanisms by which obesity influences the development of breast cancer are complex and multifaceted. This paper reviews the endocrine mechanisms, the development of the obesity-related inflammatory and immunosuppressive microenvironments, the intestinal microbiota balance, and biological clock homeostasis. A comprehensive understanding of these mechanisms can provide a theoretical foundation for diagnosing and treating obesity-related breast cancer.

The mechanisms through which obesity influences breast cancer are not isolated but rather form an interconnected network: Adipokine imbalance induced by obesity not only acts on tumor cells via pathways such as JAK/STAT3 but also modulates the immune microenvironment by regulating macrophage polarization. Gut microbiota dysbiosis affects estrogen metabolism through β-glucuronidase, and its metabolites can further influence the stemness of immune cells. Polymorphisms in obesity-related genes interact with circadian rhythm disruption, where the former may affect the expression of clock genes, while the latter exacerbates metabolic and inflammatory abnormalities. These mechanisms are coupled through key nodes such as inflammatory factors, metabolic pathways, and immune regulation, collectively driving the progression of obesity-associated breast cancer.

Future studies should integrate basic, clinical, and translational research through multidisciplinary collaboration to develop effective interventions aimed at improving the overall prognosis of individuals with obesity-related breast cancer.

## Data Availability

Data sharing is not applicable to this article as no datasets were generated or analysed during the current study.

## References

[1] Hurtado MD, Tama E, D’Andre S, et al. The relation between excess adiposity and breast cancer in women: clinical implications and management. Crit Rev Oncol/Hematol. 2024;193:104213. 38008197 10.1016/j.critrevonc.2023.104213 PMC10843740

[2] McLachlan S, NCD Risk Factor Collaboration. Trends in adult body mass index in 200 countries since 1975: pooled analysis of 1,698 population-based measurement studies with 19.2 million participants. Lancet, 2016, 387(10026). 10.1016/S0140-6736(16)30054-X PMC7615134 27115820

[3] Lohmann AE, Goodwin PJ, Chlebowski RT, et al. Association of obesity-related metabolic disruptions with cancer risk and outcome. J Clin Oncol. 2016;34(35):4249–55. 27903146 10.1200/JCO.2016.69.6187

[4] Kim JW, Kim JH, Lee YJ. The role of adipokines in tumor progression and its association with obesity. Biomedicines. 2024;12(1):97. 38255203 10.3390/biomedicines12010097 PMC10813163

[5] Tran TXM, Chang Y, Choi HR, et al. Adiposity, body composition Measures, and breast cancer risk in Korean premenopausal Women. JAMA Netw Open. 2024;7(4):e245423–245423. 38578637 10.1001/jamanetworkopen.2024.5423 PMC10998159

[6] Wang X, Simpson ER, Brown KA. Aromatase overexpression in dysfunctional adipose tissue links obesity to postmenopausal breast cancer. J Steroid Biochem Mol Biol. 2015;153:35–44. 26209254 10.1016/j.jsbmb.2015.07.008

[7] Laudisio D, Muscogiuri G, Barrea L, et al. Obesity and breast cancer in premenopausal women: current evidence and future perspectives. Eur J Obstet Gynecol Reproductive Biology. 2018;230:217–21. 10.1016/j.ejogrb.2018.03.050 29730021

[8] Kaul K, Misri S, Ramaswamy B, et al. Contribution of the tumor and obese microenvironment to triple negative breast cancer. Cancer Lett. 2021;509:115–20. 33798632 10.1016/j.canlet.2021.03.024

[9] D’Esposito V, Liguoro D, Ambrosio MR, et al. Adipose microenvironment promotes triple negative breast cancer cell invasiveness and dissemination by producing CCL5. Oncotarget. 2016;7(17):24495. 27027351 10.18632/oncotarget.8336 PMC5029717

[10] Coelho M, Oliveira T, Fernandes R. State of the Art paper biochemistry of adipose tissue: an endocrine organ. Archives Med Sci. 2013;9(2):191–200. 10.5114/aoms.2013.33181 PMC3648822 23671428

[11] Divella R, De Luca R, Abbate I, et al. Obesity and cancer: the role of adipose tissue and adipo-cytokines-induced chronic inflammation. J Cancer. 2016;7(15):2346. 27994674 10.7150/jca.16884 PMC5166547

[12] Argolo DF, Hudis CA, Iyengar NM. The impact of obesity on breast cancer. Curr Oncol Rep. 2018;20:1–8. 29644507 10.1007/s11912-018-0688-8

[13] Garofalo C, Koda M, Cascio S, et al. Increased expression of leptin and the leptin receptor as a marker of breast cancer progression: possible role of obesity-related stimuli. Clin Cancer Res. 2006;12(5):1447–53. 16533767 10.1158/1078-0432.CCR-05-1913

[14] Pan H, Deng LL, Cui JQ, et al. Association between serum leptin levels and breast cancer risk: an updated systematic review and meta-analysis. Medicine. 2018;97(27):e11345. 29979411 10.1097/MD.0000000000011345 PMC6076146

[15] Schoemaker MJ, Nichols HB, Wright LB, et al. Association of body mass index and age with subsequent breast cancer risk in premenopausal women. JAMA Oncol. 2018;4(11):e181771–181771. 29931120 10.1001/jamaoncol.2018.1771 PMC6248078

[16] Buonaiuto R, Napolitano F, Parola S, et al. Insight on the role of leptin: a Bridge from obesity to breast cancer. Biomolecules. 2022;12(10):1394. 36291602 10.3390/biom12101394 PMC9599120

[17] Demaria M, Giorgi C, Lebiedzinska M, et al. A STAT3-mediated metabolic switch is involved in tumour transformation and STAT3 addiction. Aging. 2010;2(11):823. 21084727 10.18632/aging.100232 PMC3006025

[18] Wang T, Fahrmann JF, Lee H, et al. JAK/STAT3-regulated fatty acid β-oxidation is critical for breast cancer stem cell self-renewal and chemoresistance. Cell Metabol. 2018;27(1):136–50. e5. 10.1016/j.cmet.2017.11.001 PMC5777338 29249690

[19] Yuan HJ, Sun KW, Yu K. Leptin promotes the proliferation and migration of human breast cancer through the extracellular-signal regulated kinase pathway. Mol Med Rep. 2014;9(1):350–4. 24213635 10.3892/mmr.2013.1786

[20] Wei L, Li K, Pang X, et al. Leptin promotes epithelial-mesenchymal transition of breast cancer via the upregulation of pyruvate kinase M2. J Experimental Clin Cancer Res. 2016;35:1–10. 10.1186/s13046-016-0446-4 PMC5073421 27769315

[21] Batra A, Okur B, Glauben R, et al. Leptin: a critical regulator of CD4 + T-cell polarization in vitro and in vivo. Endocrinology. 2010;151(1):56–62. 19966187 10.1210/en.2009-0565

[22] Carbone F, La Rocca C, Matarese G. Immunological functions of leptin and adiponectin. Biochimie. 2012;94(10):2082–8. 22750129 10.1016/j.biochi.2012.05.018

[23] Zhou Y, Yu X, Chen H, et al. Leptin deficiency shifts mast cells toward anti-inflammatory actions and protects mice from obesity and diabetes by polarizing M2 macrophages. Cell Metabol. 2015;22(6):1045–58. 10.1016/j.cmet.2015.09.013 PMC4670585 26481668

[24] García-Estevez L, González-Martínez S, Moreno-Bueno G. The leptin axis and its association with the adaptive immune system in breast cancer. Front Immunol. 2021;12:784823. 34868066 10.3389/fimmu.2021.784823 PMC8634160

[25] Nehme R, Diab-Assaf M, Decombat C, et al. Targeting adiponectin in breast cancer. Biomedicines. 2022;10(11):2958. 36428526 10.3390/biomedicines10112958 PMC9687473

[26] Nguyen TMD. Adiponectin: role in physiology and pathophysiology. Int J Prev Med. 2020;11(1):136. 33088464 10.4103/ijpvm.IJPVM_193_20 PMC7554603

[27] Gui Y, Pan Q, Chen X, et al. The association between obesity related adipokines and risk of breast cancer: a meta-analysis. Oncotarget. 2017;8(43):75389. 29088874 10.18632/oncotarget.17853 PMC5650429

[28] Gu L, Cao C, Fu J, et al. Serum adiponectin in breast cancer: A meta-analysis. Medicine. 2018;97(29):e11433. 30024516 10.1097/MD.0000000000011433 PMC6086546

[29] Mallik R, Chowdhury TA. Metformin in cancer. Diabetes Res Clin Pract. 2018;143:409–19. 29807101 10.1016/j.diabres.2018.05.023

[30] Alanteet AA, Attia HA, Shaheen S, et al. Anti-proliferative activity of glucagon-like peptide-1 receptor agonist on obesity-associated breast cancer: the impact on modulating adipokines’expression in adipocytes and cancer cells. Dose-Response. 2021;19(1):1559325821995651. 33746653 10.1177/1559325821995651 PMC7903831

[31] Liu J, Lam JBB, Chow KHM, et al. Adiponectin stimulates Wnt inhibitory factor-1 expression through epigenetic regulations involving the transcription factor specificity protein 1. Carcinogenesis. 2008;29(11):2195–202. 18701434 10.1093/carcin/bgn194

[32] Dieudonne MN, Bussiere M, Dos Santos E, et al. Adiponectin mediates antiproliferative and apoptotic responses in human MCF7 breast cancer cells. Biochem Biophys Res Commun. 2006;345(1):271–9. 16678125 10.1016/j.bbrc.2006.04.076

[33] Jardé T, Caldefie-Chézet F, Goncalves-Mendes N, et al. Involvement of adiponectin and leptin in breast cancer: clinical and in vitro studies. Endocrine-related Cancer. 2009;16(4):1197–210. 19661131 10.1677/ERC-09-0043

[34] Monks M, Irakleidis F, Tan PH. Complex interaction of adiponectin-mediated pathways on cancer treatment: a novel therapeutic target. Journal of Cancer Metastasis and Treatment, 2019, 5: N/A-N/A.

[35] Lu Y, Hajjar A, Cryns VL, et al. Breast cancer risk for women with diabetes and the impact of metformin: a meta-analysis. Cancer Med. 2023;12(10):11703–18. 36533539 10.1002/cam4.5545 PMC10242307

[36] Luís C, Dias J, Firmino-Machado J, et al. A retrospective study in tumour characteristics and clinical outcomes of overweight and obese women with breast cancer. Breast Cancer Res Treat. 2023;198(1):89–101. 36576677 10.1007/s10549-022-06836-5 PMC9883351

[37] Pati S, Irfan W, Jameel A, et al. Obesity and cancer: a current overview of epidemiology, pathogenesis, outcomes, and management. Cancers. 2023;15(2):485. 36672434 10.3390/cancers15020485 PMC9857053

[38] Calip GS, Yu O, Boudreau DM, et al. Diabetes and differences in detection of incident invasive breast cancer. Cancer Causes Control. 2019;30:435–41. 30949885 10.1007/s10552-019-01166-2 PMC6467528

[39] Fernández-Arce L, Robles-Rodríguez N, Fernández-Feito A, et al. Type 2 diabetes and all-cause mortality among Spanish women with breast cancer. Cancer Causes Control. 2022;33(2):271–8. 34853980 10.1007/s10552-021-01526-x PMC8776668

[40] McTiernan A. Diet and prognosis in women with breast cancer. Cancer Epidemiol Biomarkers Prev. 2021;30(2):252–4. 33547146 10.1158/1055-9965.EPI-20-1506

[41] Huang Y, Cai X, Qiu M, et al. Prediabetes and the risk of cancer: a meta-analysis. Diabetologia. 2014;57:2261–9. 25208757 10.1007/s00125-014-3361-2

[42] Cohen DH, LeRoith D, Obesity. Type 2 diabetes, and cancer: the insulin and IGF connection. Endocrine-related Cancer. 2012;19(5):F27–45. 22593429 10.1530/ERC-11-0374

[43] Kallamadi PR, Esari D, Addi UR, et al. Obesity associated with prediabetes increases the risk of breast cancer development and Progression—A study on an obese rat model with impaired glucose Tolerance. Int J Mol Sci. 2023;24(14):11441. 37511200 10.3390/ijms241411441 PMC10380482

[44] Kalluri R. The biology and function of exosomes in cancer. J Clin Investig. 2016;126(4):1208–15. 27035812 10.1172/JCI81135 PMC4811149

[45] e W, Deng WW, Zan M, et al. Cancer cell membrane camouflaged nanoparticles to realize starvation therapy together with checkpoint Blockades for enhancing cancer therapy. ACS Nano. 2019;13(3):2849–57. 30803232 10.1021/acsnano.8b03788

[46] Costa-Silva B, Aiello NM, Ocean AJ, et al. Pancreatic cancer exosomes initiate pre-metastatic niche formation in the liver. Nat Cell Biol. 2015;17(6):816–26. 25985394 10.1038/ncb3169 PMC5769922

[47] Tysoe O. Adipocyte-derived exosomes drive cancer metastasis. Nat Reviews Endocrinol. 2022;18(2):68–68. 10.1038/s41574-021-00622-x 34916665

[48] Jafari N, Kolla M, Meshulam T, et al. Adipocyte-derived exosomes May promote breast cancer progression in type 2 diabetes. Sci Signal. 2021;14(710):eabj2807. 34813359 10.1126/scisignal.abj2807 PMC8765301

[49] Llevenes P, Chen A, Lawton M et al. Plasma exosomes in insulin resistant obesity exacerbate progression of triple negative breast Cancer. bioRxiv, 2024: 2024.10. 10.617639. 10.1186/s12885-025-14447-8 PMC12239313 40629272

[50] La Camera G, Gelsomino L, Malivindi R, et al. Adipocyte-derived extracellular vesicles promote breast cancer cell malignancy through HIF-1α activity. Cancer Lett. 2021;521:155–68. 34425186 10.1016/j.canlet.2021.08.021

[51] Sadegh-Nejadi S, Afrisham R, Emamgholipour S, et al. Influence of plasma Circulating exosomes obtained from obese women on tumorigenesis and Tamoxifen resistance in MCF‐7 cells. IUBMB Life. 2020;72(9):1930–40. 32542981 10.1002/iub.2305

[52] Esmaeili F, Abolhasani M, Zabihi-Mahmoudabadi H, et al. Exosomes isolated from metabolically unhealthy normal weight and overweight phenotypes deteriorate the ER/PR positive breast cancer behavior. J Diabetes Metabolic Disorders. 2024;23(1):533–44. 10.1007/s40200-023-01295-1 PMC11196455 38932828

[53] Abdullaev B, Rasyid SA, Ali E, et al. Effective exosomes in breast cancer: focusing on diagnosis and treatment of cancer progression. Pathology-Research Pract. 2024;253:154995. 10.1016/j.prp.2023.154995 38113765

[54] Longo M, Zatterale F, Naderi J, et al. Adipose tissue dysfunction as determinant of obesity-associated metabolic complications. Int J Mol Sci. 2019;20(9):2358. 31085992 10.3390/ijms20092358 PMC6539070

[55] Kolb R, Zhang W. Obesity and breast cancer: a case of inflamed adipose tissue. Cancers. 2020;12(6):1686. 32630445 10.3390/cancers12061686 PMC7352736

[56] Zatterale F, Longo M, Naderi J, et al. Chronic adipose tissue inflammation linking obesity to insulin resistance and type 2 diabetes. Front Physiol. 2020;10:1607. 32063863 10.3389/fphys.2019.01607 PMC7000657

[57] Trayhurn P. Hypoxia and adipose tissue function and dysfunction in obesity. Physiol Rev. 2013;93(1):1–21. 23303904 10.1152/physrev.00017.2012

[58] Han JM, Levings MK. Immune regulation in obesity-associated adipose inflammation. J Immunol. 2013;191(2):527–32. 23825387 10.4049/jimmunol.1301035

[59] Faria SS, Corrêa LH, Heyn GS, et al. Obesity and breast cancer: the role of crown-like structures in breast adipose tissue in tumor progression, prognosis, and therapy. J Breast Cancer. 2020;23(3):233. 32595986 10.4048/jbc.2020.23.e35 PMC7311368

[60] Hachiya R, Tanaka M, Itoh M, et al. Molecular mechanism of crosstalk between immune and metabolic systems in metabolic syndrome. Inflamm Regeneration. 2022;42(1):13. 10.1186/s41232-022-00198-7 PMC9057063 35490239

[61] Vallega KA, Bosco DB, Ren Y, et al. Macrophage-conditioned media promotes adipocyte cancer association, which in turn stimulates breast cancer proliferation and migration. Biomolecules. 2022;12(12):1757. 36551185 10.3390/biom12121757 PMC9775594

[62] Blaszczak AM, Jalilvand A, Hsueh WA. Adipocytes, innate immunity and obesity: A mini-review. Front Immunol. 2021;12:650768. 34248937 10.3389/fimmu.2021.650768 PMC8264354

[63] Mikhailova SV, Ivanoshchuk DE. Innate-immunity genes in obesity. J Personalized Med. 2021;11(11):1201. 10.3390/jpm11111201 PMC8623883 34834553

[64] Lamabadusuriya DA, Jayasena H, Bopitiya AK, et al. Obesity-driven inflammation and cancer risk: a comprehensive review[C]//Seminars in cancer biology. Academic; 2025. 10.1016/j.semcancer.2025.07.007 40714142

[65] Tripathi D, Kant S, Pandey S, et al. Resistin in metabolism, inflammation, and disease. FEBS J. 2020;287(15):3141–9. 32255270 10.1111/febs.15322

[66] Fuentes-Mattei E, Velazquez-Torres G, Phan L, et al. Effects of obesity on transcriptomic changes and cancer hallmarks in Estrogen receptor–positive breast cancer. JNCI: J Natl Cancer Inst. 2014;106(7):dju158. 24957076 10.1093/jnci/dju158 PMC4110474

[67] Harris MA, Savas P, Virassamy B, et al. Towards targeting the breast cancer immune microenvironment. Nat Rev Cancer. 2024;24(8):554–77. 38969810 10.1038/s41568-024-00714-6

[68] Nishimura S, Manabe I, Nagasaki M, et al. CD8 + effector T cells contribute to macrophage recruitment and adipose tissue inflammation in obesity. Nat Med. 2009;15(8):914–20. 19633658 10.1038/nm.1964

[69] Jayasingam SD, Citartan M, Thang TH, et al. Evaluating the polarization of tumor-associated macrophages into M1 and M2 phenotypes in human cancer tissue: technicalities and challenges in routine clinical practice. Front Oncol. 2020;9:1512. 32039007 10.3389/fonc.2019.01512 PMC6992653

[70] Wang Y, Zhang X, **e X, et al. Obesity and metabolic syndrome related macrophage promotes PD-L1 expression in TNBC through IL6/JAK/STAT pathway and can be reversed by telmisartan. Cancer Biol Ther. 2020;21(12):1179–90. 33218268 10.1080/15384047.2020.1838032 PMC7722790

[71] Tiwari P, Blank A, Cui C, et al. Metabolically activated adipose tissue macrophages link obesity to triple-negative breast cancer. J Exp Med. 2019;216(6):1345–58. 31053611 10.1084/jem.20181616 PMC6547867

[72] Feuerer M, Herrero L, Cipolletta D, et al. Lean, but not obese, fat is enriched for a unique population of regulatory T cells that affect metabolic parameters. Nat Med. 2009;15(8):930–9. 19633656 10.1038/nm.2002 PMC3115752

[73] Wang Z, Aguilar EG, Luna JI, et al. Paradoxical effects of obesity on T cell function during tumor progression and PD-1 checkpoint blockade. Nat Med. 2019;25(1):141–51. 30420753 10.1038/s41591-018-0221-5 PMC6324991

[74] Reilly SM, Saltiel AR. Adapting to obesity with adipose tissue inflammation. Nat Reviews Endocrinol. 2017;13(11):633–43. 10.1038/nrendo.2017.90 28799554

[75] Deng T, Lyon CJ, Bergin S, et al. Obesity, inflammation, and cancer. Annu Rev Pathol. 2016;11(1):421–49. 27193454 10.1146/annurev-pathol-012615-044359

[76] Facciabene A, Motz GT, Coukos G. T-regulatory cells: key players in tumor immune escape and angiogenesis. Cancer Res. 2012;72(9):2162–71. 22549946 10.1158/0008-5472.CAN-11-3687 PMC3342842

[77] Laue T, Wrann CD, Hoffmann-Castendiek B, et al. Altered NK cell function in obese healthy humans. BMC Obes. 2015;2:1–10. 26217516 10.1186/s40608-014-0033-1 PMC4511543

[78] Wrann CD, Laue T, Hübner L, et al. Short-term and long-term leptin exposure differentially affect human natural killer cell immune functions. Am J Physiology-Endocrinology Metabolism. 2012;302(1):E108–16. 10.1152/ajpendo.00057.2011 21952038

[79] Chen J, Douglass J, Prasath V, et al. The Microbiome and breast cancer: a review. Breast Cancer Res Treat. 2019;178:493–6. 31456069 10.1007/s10549-019-05407-5

[80] Chen M, Li S, Arora I, et al. Maternal soybean diet on prevention of obesity-related breast cancer through early-life gut Microbiome and epigenetic regulation. J Nutr Biochem. 2022;110:109119. 35933021 10.1016/j.jnutbio.2022.109119 PMC9792070

[81] Vallianou N, Stratigou T, Christodoulatos GS, et al. Understanding the role of the gut Microbiome and microbial metabolites in obesity and obesity-associated metabolic disorders: current evidence and perspectives. Curr Obes Rep. 2019;8:317–32. 31175629 10.1007/s13679-019-00352-2

[82] Chen Y, Zhou J, Wang L. Role and mechanism of gut microbiota in human disease. Front Cell Infect Microbiol. 2021;11:625913. 33816335 10.3389/fcimb.2021.625913 PMC8010197

[83] Alpuim Costa D, Nobre JG, Batista MV, et al. Human microbiota and breast cancer—is there any relevant link?—a literature review and new horizons toward personalised medicine. Front Microbiol. 2021;12:584332. 33716996 10.3389/fmicb.2021.584332 PMC7947609

[84] Parida S, Wu S, Siddharth S et al. A procarcinogenic colon microbe promotes breast tumorigenesis and metastatic progression and concomitantly activates notch and β-catenin axes. Cancer Discov. 2021; 11: 1138–1157. doi: 10.1158/2159-8290. . CD-20-0537. PMID: https://www.ncbi.nlm.nih.gov/pubmed/33408241, 1138–1157. 10.1158/2159-8290.CD-20-0537 33408241

[85] Reytor-González C, Simancas-Racines D, Román-Galeano NM, et al. Obesity and breast cancer: exploring the nexus of chronic inflammation, metabolic dysregulation, and nutritional strategies. Food Agricultural Immunol. 2025;36(1):2521270.

[86] Sui Y, Wu J, Chen J. The role of gut microbial β-glucuronidase in Estrogen reactivation and breast cancer. Front Cell Dev Biology. 2021;9:631552. 10.3389/fcell.2021.631552 PMC8388929 34458248

[87] Yende AS, Sharma D. Obesity, dysbiosis and inflammation: interactions that modulate the efficacy of immunotherapy. Front Immunol. 2024;15:1444589. 39253073 10.3389/fimmu.2024.1444589 PMC11381382

[88] Chen J, Liu X, Zou Y et al. A high-fat diet promotes cancer progression by inducing gut microbiota–mediated leucine production and PMN-MDSC differentiation. Proceedings of the National Academy of Sciences, 2024, 121(20): e2306776121. 10.1073/pnas.2306776121 PMC11098111 38709933

[89] Roy S, Trinchieri G. Microbiota: a key orchestrator of cancer therapy. Nat Rev Cancer. 2017;17(5):271–85. 28303904 10.1038/nrc.2017.13

[90] Di Modica M, Gargari G, Regondi V, et al. Gut microbiota condition the therapeutic efficacy of trastuzumab in HER2-positive breast cancer. Cancer Res. 2021;81(8):2195–206. 33483370 10.1158/0008-5472.CAN-20-1659

[91] Jia D, Wang Q, Qi Y, et al. Microbial metabolite enhances immunotherapy efficacy by modulating T cell stemness in pan-cancer. Cell. 2024;187(7):1651–65. e21. 38490195 10.1016/j.cell.2024.02.022

[92] Feng ZP, **n HY, Zhang ZW, et al. Gut microbiota homeostasis restoration May become a novel therapy for breast cancer. Investig New Drugs. 2021;39(3):871–8. 33454868 10.1007/s10637-021-01063-z

[93] Simone V, D’avenia M, Argentiero A, et al. Obesity and breast cancer: molecular interconnections and potential clinical applications. Oncologist. 2016;21(4):404–17. 26865587 10.1634/theoncologist.2015-0351 PMC4828118

[94] Li Y, Su R, Deng X, et al. FTO in cancer: functions, molecular mechanisms, and therapeutic implications. Trends Cancer. 2022;8(7):598–614. 35346615 10.1016/j.trecan.2022.02.010

[95] Varghese A, Waheed SO, Chaturvedi SS et al. Revealing the catalytic strategy of FTO. Chem Catal, 2023, 3(9). 10.1016/j.checat.2023.100732 PMC12240458 40636456

[96] Ajabnoor GMA. The molecular and genetic interactions between obesity and breast cancer risk. Medicina. 2023;59(7):1338. 37512149 10.3390/medicina59071338 PMC10384495

[97] Gholamalizadeh M, Jarrahi AM, Akbari ME, et al. Association between FTO gene polymorphisms and breast cancer: the role of estrogen. Expert Rev Endocrinol Metabolism. 2020;15(2):115–21. 10.1080/17446651.2020.1730176 32089015

[98] Snoussi K, Strosberg AD, Bouaouina N, et al. Leptin and leptin receptor polymorphisms are associated with increased risk and poor prognosis of breast carcinoma. BMC Cancer. 2006;6(1):38. 16504019 10.1186/1471-2407-6-38 PMC1397853

[99] Cleveland RJ, Gammon MD, Long CM, et al. Common genetic variations in the LEP and LEPR genes, obesity and breast cancer incidence and survival. Breast Cancer Res Treat. 2010;120(3):745–52. 19697123 10.1007/s10549-009-0503-1 PMC3571680

[100] Zeng T, Zhao J, Kang Y, et al. Association between polymorphism near the MC4R gene and cancer risk: A meta-analysis. Medicine. 2020;99(36):e22003. 32899047 10.1097/MD.0000000000022003 PMC7478401

[101] Baroni MG, Arca M, Sentinelli F, et al. The G972R variant of the insulin receptor substrate-1 (IRS-1) gene, body fat distribution and insulin-resistance. Diabetologia. 2001;44(3):367–72. 11317670 10.1007/s001250051628

[102] Duggan C, Baumgartner RN, Baumgartner KB, et al. Genetic variation in TNFα, PPARγ, and IRS-1 genes, and their association with breast-cancer survival in the HEAL cohort. Breast Cancer Res Treat. 2018;168(2):567–76. 29256014 10.1007/s10549-017-4621-x PMC5839976

[103] Liu F, Chang HC. Physiological links of circadian clock and biological clock of aging. Protein Cell. 2017;8(7):477–88. 28108951 10.1007/s13238-016-0366-2 PMC5498335

[104] Touitou Y, Reinberg A, Touitou D. Association between light at night, melatonin secretion, sleep deprivation, and the internal clock: health impacts and mechanisms of circadian disruption. Life Sci. 2017;173:94–106. 28214594 10.1016/j.lfs.2017.02.008

[105] Parsons MJ, Moffitt TE, Gregory AM, et al. Social jetlag, obesity and metabolic disorder: investigation in a cohort study. Int J Obes. 2015;39(5):842–8. 10.1038/ijo.2014.201 PMC4422765 25601363

[106] Turek FW, Joshu C, Kohsaka A, et al. Obesity and metabolic syndrome in circadian clock mutant mice. Science. 2005;308(5724):1043–5. 15845877 10.1126/science.1108750 PMC3764501

[107] Pan X, Jiang XC, Hussain MM. Impaired cholesterol metabolism and enhanced atherosclerosis in clock mutant mice. Circulation. 2013;128(16):1758–69. 24014832 10.1161/CIRCULATIONAHA.113.002885 PMC3897228

[108] Karlsson B, Knutsson A, Lindahl B. Is there an association between shift work and having a metabolic syndrome? Results from a population based study of 27 485 people. Occup Environ Med. 2001;58(11):747–52. 11600731 10.1136/oem.58.11.747 PMC1740071

[109] Wang S, Lin Y, Gao L, et al. PPAR-γ integrates obesity and adipocyte clock through epigenetic regulation of Bmal1. Theranostics. 2022;12(4):1589. 35198059 10.7150/thno.69054 PMC8825584

[110] Grosbellet E, Dumont S, Schuster-Klein C, et al. Circadian phenoty** of obese and diabetic db/db mice. Biochimie. 2016;124:198–206. 26144489 10.1016/j.biochi.2015.06.029

[111] Tahira K, Ueno T, Fukuda N, et al. Obesity alters the expression profile of clock genes in peripheral blood mononuclear cells. Preliminary results. Archives Med Sci. 2011;7(6):933–40. 10.5114/aoms.2011.26603 PMC3264983 22328874

[112] Pivovarova O, Gögebakan Ö, Sucher S, et al. Regulation of the clock gene expression in human adipose tissue by weight loss. Int J Obes. 2016;40(6):899–906. 10.1038/ijo.2016.34 26902807

[113] Yan Y, Su L, Huang S, et al. Circadian rhythms and breast cancer: unraveling the biological clock’s role in tumor microenvironment and ageing. Front Immunol. 2024;15:1444426. 39139571 10.3389/fimmu.2024.1444426 PMC11319165

[114] De Visser KE, Joyce JA. The evolving tumor microenvironment: from cancer initiation to metastatic outgrowth. Cancer Cell. 2023;41(3):374–403. 36917948 10.1016/j.ccell.2023.02.016

[115] Bevinakoppamath S, Ramachandra SC, Yadav AK, et al. Understanding the emerging link between circadian rhythm, Nrf2 pathway, and breast cancer to overcome drug resistance. Front Pharmacol. 2022;12:719631. 35126099 10.3389/fphar.2021.719631 PMC8807567

[116] Hadadi E, Acloque H. Role of circadian rhythm disorders on EMT and tumour–immune interactions in endocrine-related cancers. Endocrine-related Cancer. 2021;28(2):R67–80. 33446614 10.1530/ERC-20-0390

[117] Aiello I, Fedele MLM, Román F, et al. Circadian disruption promotes tumor-immune microenvironment remodeling favoring tumor cell proliferation. Sci Adv. 2020;6(42):eaaz4530. 33055171 10.1126/sciadv.aaz4530 PMC7556830

[118] Hadadi E, Taylor W, Li XM, et al. Chronic circadian disruption modulates breast cancer stemness and immune microenvironment to drive metastasis in mice. Nat Commun. 2020;11(1):3193. 32581213 10.1038/s41467-020-16890-6 PMC7314789

[119] Blakeman V, Williams JL, Meng QJ, et al. Circadian clocks and breast cancer. Breast Cancer Res. 2016;18:1–9. 27590298 10.1186/s13058-016-0743-z PMC5010688

[120] Zhao B, **n Z, Ren P, et al. The role of PPARs in breast cancer. Cells. 2022;12(1):130. 36611922 10.3390/cells12010130 PMC9818187

